# Incidence and severity of SARS-CoV-2 infection in former Q fever patients as compared to the Dutch population, 2020–2021

**DOI:** 10.1017/S0950268822001029

**Published:** 2022-06-08

**Authors:** Elisabeth Maria den Boogert, Marit M. A. de Lange, Cornelia C. H. Wielders, Ariene Rietveld, RIVM COVID-19 Surveillance and Epidemiology Team, Mirjam J. Knol, Arianne B. van Gageldonk-Lafeber

**Affiliations:** 1 Centre for Infectious Diseases, Epidemiology and Surveillance, National Institute for Public Health and the Environment (RIVM), Bilthoven, the Netherlands; 2 Department of Infectious Disease Control, Municipal Health Service Hart voor Brabant, ‘s-Hertogenbosch, the Netherlands; 3 ECDC Fellowship Programme, Field Epidemiology Path (EPIET), European Centre for Disease Prevention and Control (ECDC), Stockholm, Sweden

**Keywords:** COVID-19, epidemiology, Q fever

## Abstract

Surveillance data shows a geographical overlap between the early coronavirus disease 2019 (COVID-19) pandemic and the past Q fever epidemic (2007–2010) in the Netherlands. We investigated the relationship between past Q fever and severe acute respiratory syndrome coronavirus 2 (SARS-CoV-2) infection in 2020/2021, using a retrospective matched cohort study.

In January 2021, former Q fever patients received a questionnaire on demographics, SARS-CoV-2 test results and related hospital/intensive care unit (ICU) admissions. SARS-CoV-2 incidence with 95% confidence intervals (CI) in former Q fever patients and standardised incidence ratios (SIR) to compare to the age-standardised SARS-CoV-2 incidence in the general regional population were calculated.

Among 890 former Q fever patients (response rate: 68%), 66 had a PCR-confirmed SARS-CoV-2 infection. Of these, nine (14%) were hospitalised and two (3%) were admitted to ICU. From February to June 2020 the SARS-CoV-2 incidence was 1573/100 000 (95% CI 749–2397) in former Q fever patients and 695/100 000 in the general population (SIR 2.26; 95% CI 1.24–3.80). The incidence was not significantly higher from September 2020 to February 2021.

We found no sufficient evidence for a difference in SARS-CoV-2 incidence or an increased severity in former Q fever patients *vs.* the general population during the period with widespread SARS-CoV-2 testing availability (September 2020–February 2021). This indicates that former Q fever patients do not have a higher risk of SARS-CoV-2 infection.

## Introduction

In February 2020, severe acute respiratory syndrome coronavirus 2 (SARS-CoV-2), the virus causing coronavirus disease 2019 (COVID-19), reached the Netherlands and started spreading. SARS-CoV-2 is a respiratory virus, with symptoms varying from mild respiratory symptoms to severe pneumonia and death [1]. From 1 February 2020 to 31 January 2021, 997 603 SARS-CoV-2 positive cases were reported in the Netherlands [2].

The COVID-19 epidemic started in several regions in the Eastern part of the province Noord-Brabant in the Netherlands. These were the same regions where a large Q fever epidemic occurred from 2007 to 2010. A preliminary study on a small group of COVID-19-related hospital admissions (mostly inhabitants of Noord-Brabant) showed a slightly higher seroprevalence of Q fever (16%) than previously estimated for high-risk areas (12–15%) [3].

Q fever is a disease caused by the bacterium *Coxiella burnetii*, which can cause symptoms ranging from mild respiratory complaints to severe pneumonia and death. However, most infected people remain asymptomatic (60%) [4]. An acute infection, if untreated, can lead to chronic Q fever in 2–5% of Q fever patients [5, 6] or long-term fatigue complaints in about 20% of Q fever patients [4].

The Q fever epidemic from 2007 to 2010 was most severe in the South-East region of the Netherlands, with a peak in cases each spring. In total, over 4000 acute Q fever cases were reported in this period. This is an underestimation of the true incidence, as not all Q fever infections were detected or reported [7]. There was a clear epidemiological link with the high density of goat farms in the affected area, and extensive veterinary measures were taken to reduce the number of Q fever cases and control the epidemic [4]. Since 2014, reports of acute Q fever cases have fallen and lie within the range of reported cases before the Q fever epidemic i.e. 1–32 annually before 2007; 7–28 annually from 2014 to 2020 [4, 8].

As surveillance data show a geographical overlap between the regions of the Q fever epidemic in 2007–2010 and the first wave of the COVID-19 epidemic in 2020 [9], we performed a retrospective cohort study to investigate whether former Q fever patients are more at risk for a SARS-CoV-2 infection and whether they are at risk for more severe COVID-19.

## Methods

A retrospective matched cohort study was performed using newly collected data on COVID-19 among patients who were diagnosed with acute Q fever in 2007–2009. Additionally, we used mandatory notification data, collected from the notification data base called OSIRIS, of SARS-CoV-2 patients reported from February 2020 to February 2021 who lived in the same region. During the first period of the COVID-19 epidemic in the Netherlands, a strict policy governed who was tested, as test capacity was limited. Not everyone with symptoms was able to get tested. From the first of June 2020, however, all inhabitants of the Netherlands could get a PCR test (and from October 2020, an antigen test as well) if they experienced symptoms that could be explained by SARS-CoV-2. To account for a potential difference in data gathered before and after the implementation of unrestricted testing, we selected two time periods. Period one was 1 February 2020–1 June 2020, and period two was 1 September 2020–1 February 2021. We omitted July and August, as the SARS-CoV-2 incidence was very low in that period.

### Former Q fever patients

The study population consisted of former Q fever patients who participated in the Q-HORT study between 2011 and 2013 [6] and who consented to further research (*n* = 1447). Q-HORT was a four-year follow-up study aiming to detect chronic Q fever cases amongst patients diagnosed with acute Q fever between 2007 and 2009. The case definition for acute Q fever is described in the study of Wielders *et al*. [6]. The questionnaire that was sent to participants gathered information on: demographic characteristics and underlying medical illness; COVID-19-related symptoms, COVID-19-related medical care (e.g. hospital or ICU admission) and SARS-CoV-2 test results for the participant or a household member. In addition, for Q-HORT participants who consented to further research during the Q-HORT study but died in 2020/2021, the Municipal Health Service (GGD Hart voor Brabant) checked whether these individuals died with a SARS-CoV-2 infection. If so, the available information on demographics, symptoms and medical care, was included in our study using the SARS-CoV-2 data from OSIRIS.

### COVID-19 in the general Dutch population

To compare the former Q fever patients to the general population, data for all persons testing positive for SARS-CoV-2 from February 2020 to February 2021 were extracted from OSIRIS. They included variables on age, gender, region, hospital and/or ICU admission and death.

**Fig. 1.**
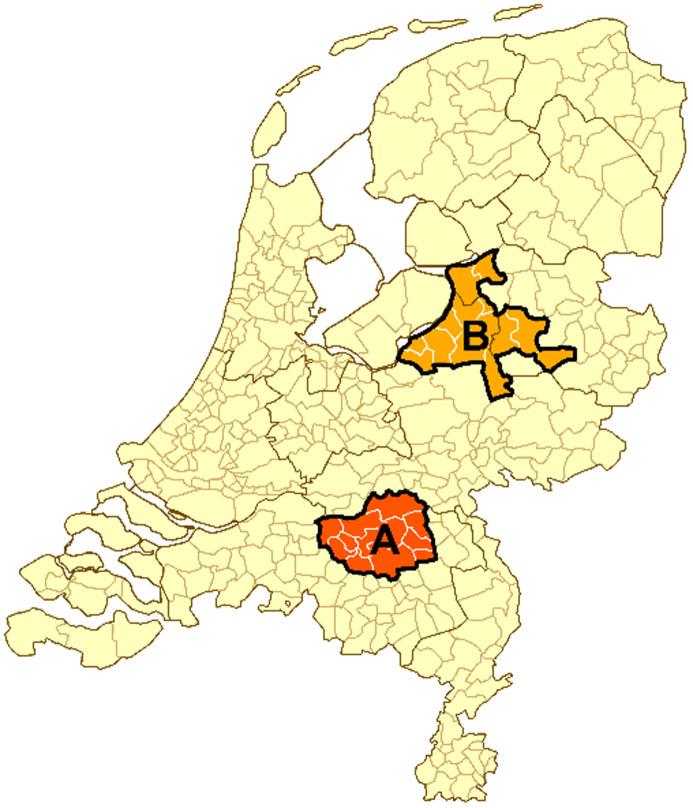
Region A includes municipalities of Q-HORT participants. Compared to Region A, Region B includes municipalities with low Q fever incidence from 2007 to 2010 but similar SARS-CoV-2 incidence from February to June 2020. (Source: OSIRIS and Statistics Netherlands (CBS)).

For comparing the SARS-CoV-2 incidence in former Q fever patients we selected Region A, which consists of 12 municipalities in the South of the Netherlands (Noord-Brabant) where most Q-HORT participants lived (Fig. 1). Age-specific SARS-CoV-2 incidences were calculated based on the mandatory case reports from Region A and were used for comparison to the former Q fever patients.

For the severity comparison we selected a second region (Region B), since COVID-19 severity might be underestimated due to more asymptomatic *C. burnetii* infections in the general population in Region A. Region B consists of 14 municipalities in the Mid-East of the Netherlands outside the area where the Q fever epidemic happened, but with a similar SARS-CoV-2 incidence from February to June 2020 as Region A (Fig. 1). To compare COVID-19 severity, former Q fever patients with self-reported laboratory-confirmed SARS-CoV-2 were matched to one case of laboratory-confirmed SARS-CoV-2 for each of the two regions. They were matched by age, gender and one of our two time periods based on date of onset or positive test result. As we selected the municipalities in Region B for their high incidence of SARS-CoV-2 infection, we did not use Region B for incidence comparison.

### Data analysis

Descriptive statistics were used to summarise patient age, gender and underlying comorbidities. SARS-CoV-2 incidences per 100 000 population were calculated, including 95% confidence intervals (CI) for incidence in the former Q fever patients. The incidence in Region A was age standardised according to the age distribution in the former Q fever patients. The SARS-CoV-2 incidence in former Q fever patients who responded by filling in the questionnaire was compared to the age-standardised SARS-CoV-2 incidence in the general population in Region A for the total study period (February 2020–February 2021), period one (February–June 2020) and period two (September 2020–February 2021). Participants of the Q-HORT study who died with SARS-CoV-2 were not included in the calculations of the SARS-CoV-2 incidence in former Q fever patients, because the data were collected differently and might have biased the calculated incidence. Furthermore, we used Poisson regression (R-package popEpi) to calculate the standardised incidence ratio (SIR) with 95% CI for SARS-CoV-2 in the former Q fever patients compared with the general population in Region A.

To determine the severity of COVID-19, conditional logistic regression (R-package survival) was conducted to compare the risk of hospitalisation, ICU admission and death between SARS-CoV-2 infections in former Q fever patients (including those who died with SARS-CoV-2) and matched SARS-CoV-2 cases from the general Dutch population living in Region A or B. For each outcome of interest, odds ratios (OR) with 95% CI were calculated. Where possible, we calculated OR and 95% CI for period one and two and Region A and B separately. Data were analysed using R 4.0.2 Statistical Software.

### Ethical approval

Former Q-HORT participants [6] who consented to further research were asked to give informed consent for the current study. It was reviewed by The Clinical Expertise Centre of the National Institute for Public Health and the Environment (RIVM) which judged that it was not subject to Act 1 of the law for Medical Research Involving Human Subjects (WMO).

## Results

Of the 1447 former Q-HORT participants, an updated Dutch address could be retrieved for 1304, of whom 890 (68%) completed the questionnaire that they received on 11 January 2021. In addition, we identified 17 Q-HORT participants who died in 2020–2021. Of these, ten were reported to the GGD Hart voor Brabant because of a laboratory-confirmed SARS-CoV-2 infection. They were therefore added to our subjects and included in the severity analysis.

Of the 900 total, 497 (55%) were male, and the median age was 63 years (54–70 interquartile range (IQR)). The majority had one or more underlying comorbidities (74.3%). The most frequently reported chronic diseases were cardiovascular disease (345/900; 38.3%), Q fever fatigue syndrome (249/890; 28.0%), chronic Q fever (122/890; 13.7%), chronic lung condition (121/900; 13.4%) and immunodeficiency (113/900; 12.6%). A small number of respondents were pregnant in 2020–2021 (5/900; 0.6%). Excluding Q fever fatigue syndrome and chronic Q fever, 63.3% of 900 former Q fever patients had one or more underlying comorbidities. Of the 900 subjects, 201 (22.3%) reported symptoms possibly related to COVID-19, but had no laboratory confirmation.

### Incidence

From February 2020 to February 2021 in Region A, the SARS-CoV-2 incidence was not significantly higher in former Q fever patients who participated in Q-HORT (*n* = 890) being 7415/100 000 (95% CI 5627–9205) compared to the age-standardised incidence of 5786/100 000 in the general population (SIR: 1.28, 95% CI 0.99–1.63, Table 1). In period one, from February to June 2020 in Region A, the SARS-CoV-2 incidence was significantly higher in former Q fever patients, being 1573/100 000 (95% CI 749–2397) compared to 695/100 000 in the general population (SIR: 2.26, 95% CI 1.4–3.80). For period two in Region A, the difference in SARS-CoV-2 incidence between former Q fever patients and the general population was not statistically significant, i.e. 5506 *vs.* 5016 (SIR: 1.10, 95% CI 0.81–1.45) (Table 1).

**Table 1.** Age-standardised incidence per 100 000 population and standardised incidence ratio of confirmed SARS-CoV-2 infection in former Q fever patients (self-reported) and the general population in Region A (Q fever region) in the Netherlands, 2020–2021

	Incidence laboratory-confirmed SARS-CoV-2 infection in former Q fever patients (95% CI)	Incidence of laboratory-confirmed SARS-CoV-2 infection in the general population	SIR (95% CI)
Total period: February 2020–February 2021	7415 (5627–9205)	5786	1.28 (0.99–1.63)
Period 1: February 2020–June 2020^a^	1573 (749–2397)	695	2.26 (1.24–3.80)
Period 2: September 2020–February 2021^b^	5506 (3964–7047)	5016	1.10 (0.81–1.45)

SIR, standardised incidence ratio; CI, confidence interval.

a SARS-CoV-2 PCR test capacity was limited during this period. Only health care workers, the elderly, persons with comorbidities and hospitalised patients could get tested.

b SARS-CoV-2 PCR (and later also antigen) tests were available for everyone with COVID-19-related symptoms.

### Severity

Of 900 former Q fever patients, 76 had a laboratory-confirmed SARS-CoV-2 infection (8.4%). Of these, 16 (21.1%) were hospitalised, five (6.6%) admitted to the ICU and ten (13.2%) died.

Of the 76, two lacked a date of onset or positive test result, and one did not match the selected time periods. The remaining 73 could be matched in age, gender and time period to two SARS-CoV-2 cases, one from Region A and one from Region B. Of the 73, 47 were male (64.4%); the median age was 63 (IQR 54–71), and 52 reported one or more underlying comorbidities (71.2%). A minority of cases received a positive test result in the first period (*n* = 21; 28.8%) compared to the second period (*n* = 52; 71.2%). The former Q fever patients showed a higher percentage of hospital admissions, ICU admissions and death (19.2%, 6.9% and 11.0%, respectively) compared to matched SARS-CoV-2 cases from the general population in Region A (16.4%, 2.7% and 8.2%, respectively) and in Region B (15.1%, 5.5% and 9.6%, respectively) (Table 2). However, the differences were not statistically significant in the conditional logistic regression analysis.

**Table 2.** Characteristics of confirmed SARS-CoV-2 infections in former Q fever patients and comparison between former Q fever patients and matched persons in the general population in Region A (Q fever region; *n* = 73), Region B (non-Q fever region; *n* = 73) and Region A and B together (*n* = 146) in the Netherlands

	Former Q fever patients		General population in Region A				General population in Region B				General population in Region A and B combined			
	*n*	%	*n*	%	OR	95% CI	*n*	%	OR	95% CI	*n*	%	OR	95% CI
**Period 1: February 2020–June 2020^a^**	21		21				21				42			
Hospitalisation	11	52.4	11	52.4	1.00	0.32–3.10	9	42.9	1.33	0.46–3.84	20	47.6	1.21	0.42–3.44
ICU	5	26.3	2	9.5	2.50	0.49–12.9	3	14.3	2.50	0.49–12.9	5	11.9	2.91	0.68–12.5
Death	6	29.6	6	28.8	1.00	0.20–4.96	5	23.8	2.00	0.18–22.1	11	26.2	1.24	0.25–6.06
**Period 2: September 2020–February 2021**^b^	52		52				52				104			
Hospitalisation	3	5.9	1	1.9	3.00	0.31–28.8	2	3.9	1.50	0.25–8.98	3	2.88	2.00	0.40–9.91
ICU	0	0	0	0	*NA*	*NA*	1	1.9	*NA*	*NA*	1	0.96	*NA*	*NA*
Death	3	5.8	0	0	*NA*	*NA*	2	3.9	1.50	0.25–8.98	2	1.92	3.00	0.50–18.0
**Total period: February 2020–February 2021**	73		73				73				146			
Hospitalisation	14	19.2	12	16.4	1.29	0.48–3.45	11	15.1	1.38	0.55–3.42	23	15.8	1.40	0.58–3.41
ICU	5	6.9	2	2.7	2.50	0.49–12.9	4	5.5	1.67	0.40–6.97	6	4.11	2.19	0.58–8.36
Death	9	12.3	6	8.2	2.00	0.50–8.00	7	9.6	1.67	0.40–6.97	13	8.90	1.85	0.56–6.16

OR, odds ratio; CI, confidence interval; ICU, intensive care unit; NA, not applicable.

a SARS-CoV-2 PCR test capacity was limited during this period. Only health care workers, the elderly, persons with comorbidities and hospitalised patients could get tested.

b SARS-CoV-2 PCR (and later also antigen) tests were available for everyone with COVID-19-related symptoms.

## Discussion

The study shows a statistically significant increase in the incidence of SARS-CoV-2 in former Q fever patients compared to the general population in Region A for period one (restricted testing policy). However, no statistically significant rise in incidence was seen for period two (large-scale testing policy) or for the entire study period from February 2020 to February 2021. In no period was there sufficient evidence for a more severe course of COVID-19 in former Q fever patients.

There are several potential explanations for the higher incidence of SARS-CoV-2 in former Q fever patients in period one. First, it could be related to the effects of acute Q fever in the past. In that case, however, we would have expected a higher incidence in the second period as well, which was not observed. Second, more testing might have occurred in former Q fever patients, especially during the first period, if their disease course was more severe compared to the general Dutch population. The matched analysis on severity outcomes (i.e. hospital admission, ICU admission and death), however, showed no such differences in either region or time period.

Finally, up to 1 June 2020 there was a restricted policy on who could get tested. More former Q fever patients had underlying illnesses (74%, or 63% if not counting QVS and chronic Q fever) than individuals in the general Dutch population (57% on 1 January 2019) [10]. Since former Q fever patients seem to have more comorbidities such as heart disease, chronic lung conditions and obesity [11, 12], they may have been more often tested for SARS-CoV-2 during the first period of the pandemic than individuals in the general population. More testing results in higher incidence. Unfortunately, the current study cannot answer the question whether the comorbidities (apart from chronic Q fever and QVS) resulted from the *C. burnetii* infection, or whether they were already present before the Q fever diagnosis, and/or whether they made these individuals more susceptible to get Q fever.

It is important to bear in mind that there is a potential bias for a higher incidence and more rapid spread of SARS-CoV-2 in Region A, where the former Q fever patients live. It has poorer air quality than other provinces in the Netherlands [13], which might have affected the incidence and disease course of SARS-CoV-2 in its former Q fever patients and the general population. A recent study found a link between poorer air quality areas in the Netherlands and the COVID-19 incidence and related hospitalisations and death [14]. Ecological studies performed in the United States of America [15], Europe [16] and the United Kingdom [17] suggest a similar relationship between air quality and COVID-19 spread and deaths. However, according to Heederik, Smit and Vermeulen [18], these studies do not fulfil quality criteria for a causal link between air pollution and COVID-19 spread, as they rely on aggregate data. In the current study, we were not able to correct for regional factors such as air quality. A national study has begun to investigate the effect of air quality on SARS-CoV-2 incidence and severity and should help to clarify their relationship [19].

## Strengths and limitations

To our knowledge, Q-HORT is the largest Q fever patient cohort worldwide that was available for follow-up. This, in combination with the high response rate to our questionnaires (68%) gives us a good overview of the COVID-19 incidence and severity in this group of patients.

However, the study has several limitations. First, the Q-HORT study group previously participated in a scientific study and consented to participate in further research, suggesting that they are a select group. Findings based on such a group may have limited application to former Q fever patients in general. Moreover, their willingness to participate could reflect a Q fever episode that was particularly severe and/or resulted in more long-term problems than usual. This applies even more to the former Q fever patients who participated in this study since they consented and participated in scientific research for a second time. Second, persons who experienced severe Q fever and/or who have long-term related problems might be more cautious and more compliant with COVID-19 measures set by the government. This could result in fewer cases of COVID-19 in former Q fever patients. Third, recall bias may have occurred, with former Q fever patients over-reporting illness episodes because they are unusually focused on health problems and symptoms. Alternatively, they might under-report episodes, having forgotten their illness. The latter is unlikely, as COVID-19 is a fairly new disease and we expect people to remember a positive test result, which we used as outcome in this study. Fourth, we cannot exclude the possibility that a former Q fever patient with a positive SARS-CoV-2 test was selected as its own match from the notification database. We were not able to identify the notified cases and compare them to the former Q fever patients due to privacy restrictions. It is highly unlikely that a former Q fever patient is its own match, as there were many SARS-CoV-2 cases, but we cannot be certain of this.

Finally, due to the low number of hospital admissions, ICU admissions and deaths, results for severity per region and time period must be interpreted with caution. Especially for period two, the conditional logistic regression could not be performed for certain subgroups due to low numbers.

## Conclusions

In general, results should be interpreted with caution given the few cases with SARS-CoV-2 in our study population. Also, other explanations exist for the higher SARS-CoV-2 incidence in period one, and further research will investigate them. The possible relationship between COVID-19 severity, air quality and livestock farms is under investigation by the RIVM [19]. Overall, we found no sufficient evidence for a difference in SARS-CoV-2 incidence or an increased severity in former Q fever patients compared to the general population during the second wave and onwards in which widespread SARS-CoV-2 testing was available (September 2020–February 2021). This indicates that former Q fever patients do not have a higher risk of SARS-CoV-2 infection.

## Data Availability

All data relevant to the study are included in the article in an aggregated and anonymised format. For legal reasons, the disaggregated dataset is available in an anonymised format from the corresponding author on reasonable request.
